# Porcine transmissible gastroenteritis virus inhibits NF-κB activity via nonstructural protein 3 to evade host immune system

**DOI:** 10.1186/s12985-019-1206-9

**Published:** 2019-08-05

**Authors:** Yanan Wang, Aoying Sun, Yu Sun, Sijia Zhang, Tian Xia, Tiantian Guo, Zhenye Hao, Li Sun, Yanping Jiang, Xinyuan Qiao, Wen Cui, Lijie Tang, Yigang Xu, Yijing Li, Li Wang

**Affiliations:** 1 Department of Preventive Veterinary Medicine, College of Veterinary Medicine, Northeast Agricultural University, Harbin, Heilongjiang China; 2 Northeastern Science Inspection Station, China Ministry of Agriculture Key Laboratory of Animal Pathogen Biology, Harbin, Heilongjiang China; 3 College of Animal Science and Technology, Northeast Agricultural University, Harbin, Heilongjiang China

**Keywords:** TGEV, Nsp3, NF-κB, Ubiquitination, PLP

## Abstract

**Background:**

Transmissible gastroenteritis virus (TGEV), a member of the family *Coronaviridae*, causes lethal watery diarrhea in piglets. Previous studies have revealed that the coronaviruses develop various strategies to evade the host innate immunity through the inhibition of nuclear factor kappa B (NF-κB) signaling pathway. However, the ability of TGEV to inhibit the host innate immune response by modulating the NF-κB signaling pathway is not clear.

**Methods:**

In this study, a dual luciferase reporter assay was used to confirm the inhibition of NF-κB by TGEV infection and to identify the major viral proteins involved in the inhibition of NF-κB signaling. Real-time quantitative PCR was used to quantify the mRNA expression of inflammatory factors. The deubiquitination of Nsp3 domains and its effect on IκBα and p65 were analyzed by western blotting. The ubiquitination level of IκBα was analyzed by immunoprecipitation.

**Results:**

In ST and IPEC-J2 cells, TGEV exhibited a dose-dependent inhibition of NF-κB activity. Individual TGEV protein screening revealed the high potential of non-structural protein 3 (Nsp3) to inhibit NF-κB signaling, and leading to the downregulation of the NF-κB-induced cytokine production. We demonstrated that the inhibitory effect of Nsp3 was mainly mediated through the suppression of IκBα degradation as well as the inhibition of p65 phosphorylation and nuclear translocation. Furthermore, the amino acid residues at positions 590–1,215 in Nsp3 were demonstrated to inhibit the degradation of IκBα by inhibiting the IκBα ubiquitination.

**Conclusion:**

TGEV infection can inhibit the activation of the NF-κB signaling pathway, which is mainly mediated by Nsp3 through the canonical pathway. The amino acid residues at positions 590–1,215 in Nsp3 compose the critical domain that mediates NF-κB inhibition. We speculate that this inhibitory effect is likely to be related to the structure of PLP2 with deubiquitinating enzyme activity of the amino acid residues at positions 590–1,215 in Nsp3. Our study provides a better understanding of the TGEV-mediated innate immune modulation and lays the basis for studies on the pathogenesis of coronavirus.

## Background

Transmissible gastroenteritis (TGE), an acute swine enteric disease, is caused by the transmissible gastroenteritis virus (TGEV). Pigs that are infected with TGEV usually present symptoms such as vomiting, dehydration, and severe diarrhea. Among the piglets aged less than 2 weeks old, the mortality rate of TGEV infection is as high as 100% [1, 2]. Globally, TGE causes enormous economic loss to the swine industry. TGEV was first identified in the United States in 1946 as an etiological agent of TGE in swine [3]. TGEV is an enveloped, positive-sense, single-stranded RNA virus with a genome size of approximately 28.6 kb. The virus belongs to the family *Coronaviridae* in the order Nidovirales [4]. The virus genome comprises 5′-untranslated region (UTR), at least nine open reading frames,and 3′-UTR [4]. ORF1 comprises two ORFs, ORF1a and ORF1b, which encode the pp1a and pp1ab polyproteins, respectively. The polyproteins are cleaved into 16 non-structural proteins (Nsp1-Nsp16) by the virus-encoded papain-like protease (PL pro) and 3C-like protease (3CL pro). These non-structural proteins have various functions in the viral life cycle [5].

The host innate immune response is the first line of defense against viral infections. Various transcription factors, such as interferon (IFN) regulatory factor 3 (IRF3), nuclear factor-κB (NF-κB), and activating transcription factor 2 (ATF-2) are activated during the immune response [6–8]. Among these transcription factors, NF-κB is the key regulator of the proinflammatory and antiviral responses. The NF-κB family comprises of five members: p65/RelA, RelB, cRel, p50, and p52. These transcription factors share an N-terminal DNA-binding/dimerization domain, which is known as the Rel homology domain. This domain plays a crucial role in the formation of homodimers and heterodimers. NF-κB dimers can bind to various target DNA sequences called κB sites and modulate the gene expression [9]. The canonical pathway for the activation of NF-κB has been extensively studied. The pathogen pattern recognition receptor on the cell surface recognizes various pro-inflammatory cytokines and pathogen molecules, resulting in the activation of the IκB kinase (IKK) complex, which is mediated by the IKKβ subunit. The phosphorylated IKKβ subunit phosphorylates the amino terminus of repressor IκB (mainly IκBα) at the Ser32 and Ser36 residues. Subsequently, repressor IκB is ubiquitinated and targeted for protein degradation by the proteolytic enzymes. The degradation of IκB exposes the nuclear localization signal (NLS), which promotes the translocation of NF-κB into the nucleus. NF-κB within the nucleus promotes the transcription of several chemokines, cytokines, and adhesion factors [9, 10].

Many viruses, such as mouse hepatitis virus (MHV), porcine reproductive respiratory syndrome virus (PRRSV), infectious bronchitis virus (IBV), and Newcastle disease virus (NDV) are known to activate the host innate immune response through NF-κB activation [11–14]. However, the virus particles may still replicate and cause disease in vivo. This suggests that the virus employs various strategies to inhibit the NF-κB signaling pathway for evading the host immune response. The Orf virus (ORFV), human immunodeficiency virus (HIV), Middle East respiratory syndrome coronavirus (MERS-CoV), and human coronavirus OC43 (HCoV-OC43) can evade the antiviral innate immunity by inhibiting the NF-κB activation [15–18]. Additionally, some viruses, such as porcine epidemic diarrhea virus (PEDV), have a dual role in the regulation of the NF-κB signaling pathway [19, 20]. As both PEDV and TGEV belong to the genus *Alphacoronavirus* within the family *Coronaviridae*, we explored whether the effect of TGEV on NF-κB signaling is similar to that of PEDV. Our previous experimental results have demonstrated that TGEV infection can activate the NF-κB and induce the production of pro-inflammatory cytokines via the NF-κB signaling pathway [21], which concurred with the results of other studies [22]. However, whether TGEV exerts an inhibitory effect on NF-κB signaling pathway is still unknown.

In this study, we demonstrated that TGEV infection exerted a dose-dependent inhibitory effect on the NF-κB signaling pathway in both intestinal epithelial cell lines J2 (IPEC-J2) and swine testicular (ST) cells. Additionally, we demonstrated that Nsp3 was the key viral protein that is involved in the regulation of NF-κB signaling through the canonical pathway and in the suppression of NF-κB-induced cytokine production. The amino acids at positions 590–1,215 in Nsp3 play a critical role in the inhibition of NF-κB signaling by inhibiting IκBα ubiquitination as well as the phosphorylation and nuclear translocation of p65. These effects appear to be related to the papain-like protease 2 (PLP2), which is located between amino acids at positions 606 and 901. Our findings provide useful insights into the mechanisms underlying the pathogenesis of coronavirus.

## Methods

### Viruses, cells, and reagents

Intestinal epithelial cell lines J2 (IPEC-J2) and HEK-293 T, were available in our laboratory. The swine testicular (ST) cells were obtained from the American Type Culture Collection (ATCC, CRL-1746). The ST cells and IPEC-J2 cells were cultured in Dulbecco’s modified Eagle’s medium (DMEM) (Gibco, 12491015, USA) supplemented with 10% fetal bovine serum (FBS) (Gibco, 10099141, USA) at 37 °C and 5% CO_2_. The TGEV TH-98 strain was isolated from the intestinal tract of TGEV-infected piglets in the Heilongjiang province of China (GenBank accession number: KU729220). The virus titer was determined using 50% tissue culture infective dose (TCID 50) assay. Mouse anti-β-actin and mouse anti-hemagglutinin (HA) monoclonal antibodies (mAbs) were purchased from Sigma (A1978, H7411, USA), while mouse mAbs against p65 and IκBα and rabbit mAbs against phospho-NF-κB p65 (6956 T, 4814 T, 3033 T, respectively) were obtained from Cell Signaling Technology (USA). The synthetic double-stranded RNA, polyinosinic:polycytidylic acid (poly(I:C)) was supplied by Sigma (P9582, USA). A cell viability assay was performed using the Cell Counting Kit-8, following the manufacturer’s instructions (Sangon Biotech, E606335–0100, China).

### Plasmids

The eukaryotic expression vectors pCMV-HA and pCMV-Myc were purchased from Clontech (635690 and 635689, respectively, Japan). The NF-κB luciferase reporter plasmid pNF-κB-Luc was supplied by Beyotime Biotechnology (D2206, China). The internal reference gene reporter plasmid pRL-TK was provided by Promega (E2241, USA). The eukaryotic expression plasmids of ubiquitin protein (Ub), TGEV-encoded proteins, and Nsp3 fragments used in this study were constructed in our laboratory. The primers used are shown in Table 1.

**Table 1:** The specific primers for Nsp3

Gene name	Primer name	Primer sequence (5′-3′)	Position (bp)
NSP3–1	Nsp3(1-418aa)-F	ACGCGTCGACCGGTGACAAAACTGTCTCATTTTCAG	1–1254
	Nsp3(1-418aa)-R	CGGCCATGGCTACTCTTTTTCAATAGAGGATTTG	
NSP3–2	Nsp3(410-601aa)-F	ACGCGTCGACCGCCAATGTAATGACTAGAGCTG	1230–1803
	Nsp3(410-601aa)-R	CGGCCATGGCTAGCTAGGCAACTGGTTTGTAACATC	
NSP3–3	Nsp3(590-1215aa)-F	ACGCGTCGACCGTCTTCGTATACACTGACCAGGAG	1770–3645
	Nsp3(590-1215aa)-R	CGGGGTACCCTA CAGAATTTACCAGTACCATTAGC	
NSP3–4	Nsp3(1168-1510aa)-F	ACGCGTCGACCCATATTGTTTTCGCATGCTCAAACCCG	3504–4510
	Nsp3(1168-1510aa)-R	CGGGGTACCCTATGAACCACTTTTTGGAGACACAG	

The restriction enzyme cutting sites are highlighted by underline

### Transfection and reporter gene assays

For TGEV infection studies, ST or IPEC-J2 cells were seeded in 24-well cell culture plates. When the cells reached a confluency of 70–80%, the cells were co-transfected with pNF-κB-luc (0.5 μg) and reference plasmid pRL-TK (0.025 μg). After 12 h, the cells were treated with poly(I:C) (10 μg/mL) or sterile phosphate-buffered saline (PBS). After 24 h, the cells were infected with TGEV. The infected cells were lysed at 12, 24, and 36 h post-infection. Firefly luciferase and *Renilla* luciferase activities were determined using a dual luciferase reporter assay system (Promega, USA), following the manufacturer’s instructions. For TGEV gene transfection studies, HEK-293 T or IPEC-J2 cells were seeded in 24-well cell culture plates. When the cells reached a confluency of 70–80%, the cells were co-transfected with pNF-κB-luc, reference plasmid pRL-TK, and either a pCMV-HA expression plasmid containing TGEV genes or an empty pCMV-HA plasmid. After 24 h, the cells were incubated with either poly(I:C) (10 μg/mL) or sterile PBS for 12 h and the cells were collected for dual luciferase activity analysis. All the values were normalized using *Renilla* luciferase activity as an internal control and expressed in terms of fold change. The data are represented as mean ± standard deviation from three independent experiments.

### RNA extraction and quantitative real-time polymerase chain reaction (RT-PCR)

The cells were washed with PBS and total cellular RNA was extracted using RNA Rapid extraction kit, following the manufacturer’s instructions (Fastagen, 220010). Total RNA was reverse transcribed into cDNA using random primers and M-MLV Reverse Transcriptase (639574, TaKaRa, Japan). The cDNA was used as a template in the SYBR Green PCR assay (Roche, Germany). The abundance of individual mRNA transcripts in each sample was assayed thrice using β-actin as an internal control. Changes in fluorescence signal throughout the reaction were detected in the ABI PRISM 7500 Real-Time PCR system. The relative transcript levels of interleukin (IL)-1, IL-6, IL-8, and tumor necrosis factor (TNF)-α were calculated according to the 2^−ΔΔCt^ threshold method. Primers are listed in Table 2.

**Table 2:** Primers used in the quantitative real-time PCR

Gene names	Forward primer sequences (5′-3′)	Reverse primer sequences (5′-3′)
IL-1	AGGGACATGGAGAAGCCATTT	TTCTCCTTGAGAGCTGATG
IL-6	AAATGCTCTTCACCTCTC	TCACACTTCTCATACTTCTC
IL-8	GTTCTGGCAAGAGTAAG	CACGGAGAATGGGTTT
TNF-α	CGTTGTAGCCAATGTCAAAGCC	TGCCCAGATTCAGCAAAGTCCA
β-actin	CCATCGTCCACCGCAAAT	CCAAATAAAGCATGCCAATC

### Western blot analysis and co-immunoprecipitation

The cells were washed with ice-cold PBS and treated with a cell lysis buffer (Beyotime, P0013G, China) containing a protease inhibitor cocktail (Sigma, P8340, USA). Nuclear and cytosolic proteins were isolated with the Nuclear and Cytoplasmic Protein Extraction Kit (Beyotime, P0027, China), following the manufacturer’s instructions. The cells were lysed on ice for 30 min, and the cellular debris was removed by centrifugation. The protein concentration in the lysate was quantified using the bicinchoninic acid (BCA) protein assay kit (Beyotime, P0011, China). The protein samples were mixed with 5X sodium dodecyl sulfate (SDS) loading buffer and boiled for 10 min. The samples were subjected to SDS polyacrylamide gel electrophoresis (PAGE) and western blotting to quantify the expression of TGEV total p65, cytoplasm IκBα, cytoplasm p-p65, and nuclear p65 using the respective antibodies. β-actin was used as the loading control.

For co-immunoprecipitation assay, IPEC-J2 and HEK-293 T cells were cultured in 100 mm dishes and transfected with pCMV-Myc-Nsp3 (590–1215 aa) and pCMV-HA-Ub for 24 h. Next, the cells were treated with 10 μg/mL poly(I:C) for 12 h. The cells were harvested and lysed with a cell lysis buffer for performing immunoprecipitation assays. MG132 (25 mM) was added to the culture medium 4 h before harvesting the cells. The samples were incubated on a plate shaker at 4 °C for 30 min. The supernatant was transferred to fresh tubes and incubated with control IgG antibody coated on agarose beads at 4 °C for 2 h. The samples were incubated with Myc monoclonal antibodies coated on agarose beads at 4 °C for 2 h. The mixture was centrifuged at 1,000 rpm and 4 °C for 1 min. The pellets were washed five times with PBS and analyzed by western blotting using HA monoclonal antibodies.

### Statistical analysis

All experiments were repeated at least three times. The experimental data were statistically analyzed with two-way repeated measure analysis of variance (RM-ANOVA) using GraphPad Prism software (version 5.0). *P*-values less than 0.05 were considered statistically significant, and those less than 0.01 were considered highly significant.

## Results

### TGEV replication inhibits NF-κB signaling

**Fig. 1:**
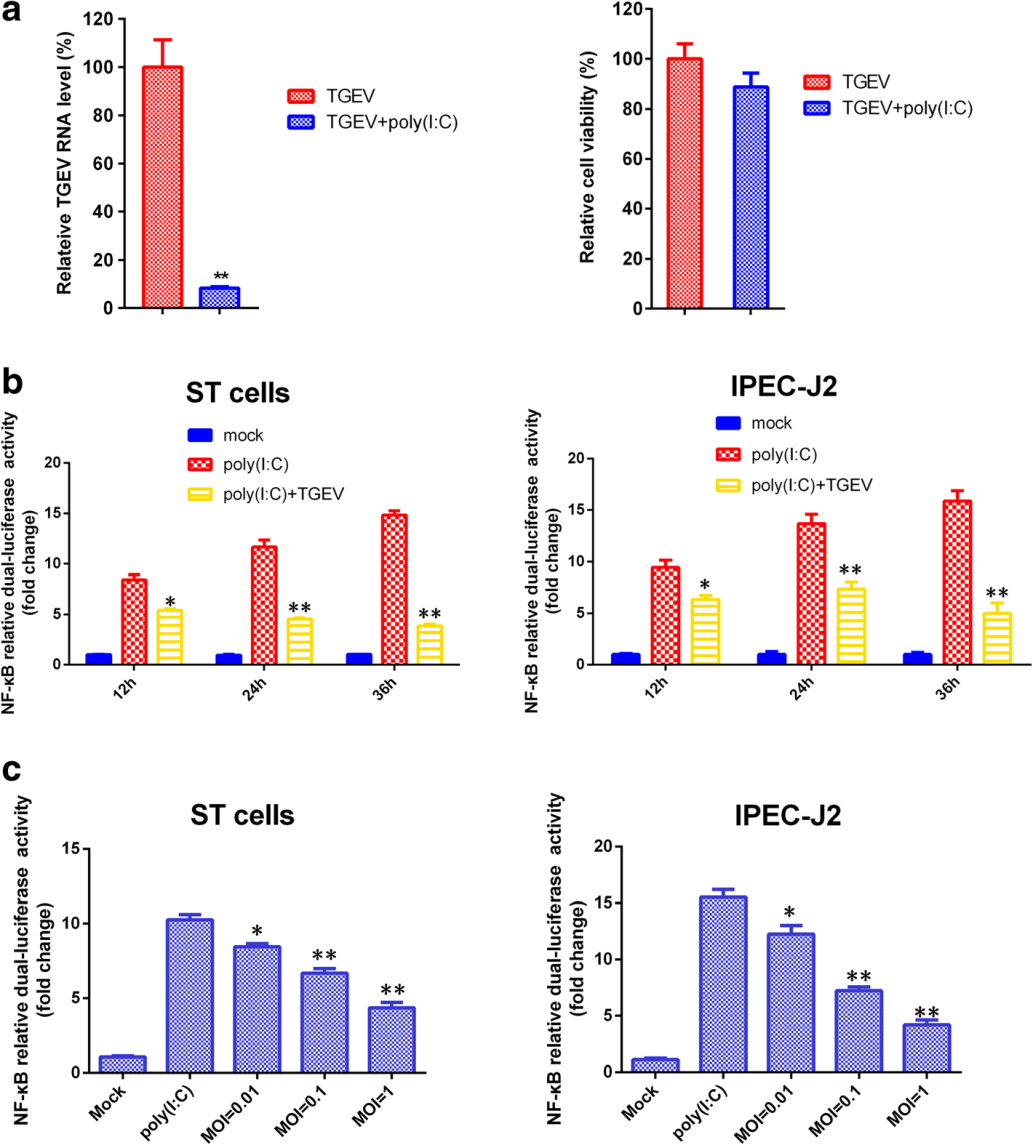
Inhibition of the NF-κB signaling pathway by TGEV replication. **a** IPEC-J2 cells were treated with 10 μg/mL poly(I:C) or sterile PBS (negative control) for 12 h. The cells were infected with TGEV at an MOI of 1. Subsequently, TGEV mRNA levels were measured by real-time PCR at 36 h post-TGEV infection, using β-actin as an internal reference gene. **b** ST cells and IPEC-J2 cells were transfected with pNF-κB-Luc (0.5 μg) and pRL-TK (0.025 μg). After 12 h, the cells were treated with 10 μg/mL poly(I:C). At 24 h post-transfection, the cells were infected with TGEV at an MOI of 1. At 12, 24, and 36 h post-TGEV infection, cell extracts were prepared for luciferase activity assay. **c** ST and IPEC-J2 cells were transfected with pNF-κB-Luc (0.5 μg) and pRL-TK (0.025 μg). After 12 h, cells were treated with poly (I:C). At 24 h post-transfection, the cells were infected with TGEV at an MOI of 0.01, 0.1, or 1 for 24 h and luciferase activity was measured. Results are representative of three independent experiments. Data are presented as mean ± standard deviation (SD). *P values < 0.05 and **P values < 0.01 were considered to be statistically significant and highly significant, respectively

Poly(I:C) is a synthetic analog of double-stranded RNA (dsRNA), which is recognized by the Toll-like Receptor 3 (TLR3). Poly(I:C) activates NF-κB signaling pathway and induces cytokine production [23]. The antiviral effect of poly(I:C) against TGEV was evaluated by treating the IPEC-J2 cells with poly(I:C) for 12 h before inoculation with the TGEV. The RT-PCR analysis revealed that poly(I:C) can significantly reduce the TGEV RNA replication, whereas its effect on cell viability was minimal (Fig. 1a). Although poly(I:C) inhibited TGEV replication in the cells, the virus could not be completely inactivated. Hence, we hypothesized that TGEV may evade the host immune system by inhibiting the poly(I:C)-activated NF-κB pathway. We co-transfected ST and IPEC-J2 cells with the pNF-κB-Luc reporter plasmid to evaluate the effect of TGEV replication on the NF-κB signaling pathway. The pRL-TK plasmid was used as an internal reference. At 12 h post-transfection, the cells were treated with poly(I:C) to induce the activation of NF-κB signaling pathway. At 24 h post-transfection, the cells were infected with TGEV at a multiplicity of infection (MOI) of 1. The infected cells were harvested for dual luciferase activity analysis at different time points. We observed that the poly(I:C)-treated group exhibited significant activation of the NF-κB signaling pathway when compared to the mock control group. However, TGEV infection resulted in time-dependent inhibition of NF-κB signaling activation (Fig. 1b). The transfected ST and IPEC-J2 cells were treated with poly(I:C) and infected with TGEV at different MOIs to evaluate the effect of viral infection titers on the inhibition of NF-κB signaling pathway. As shown in Fig. 1c, NF-κB signaling was significantly activated in the poly(I:C)-treated group when compared to the control group. TGEV infection resulted in significant dose-dependent inhibition of the NF-κB pathway activation.

### TGEV Nsp3 overexpression inhibits the NF-κB signaling pathway

**Fig. 2:**
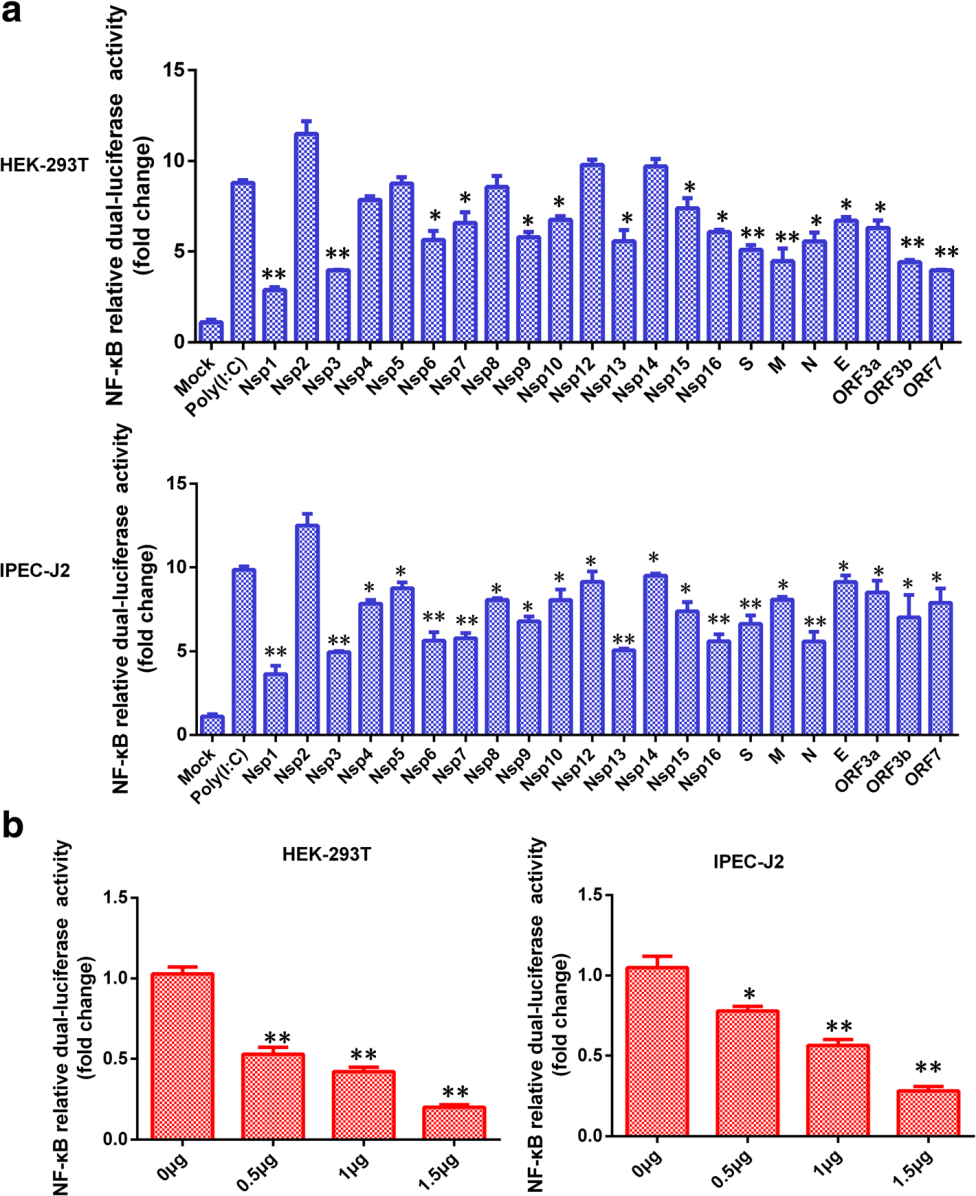
Nsp3 protein in TGEV plays a crucial role in the inhibition of NF-κB signaling pathway. **a** IPEC-J2 cells and HEK-293 T cells were co-transfected with pNF-κB-Luc (0.5 μg), pRL-TK (0.025 μg), and the respective expression plasmid encoding either the TGEV protein or truncated segments (0.5 μg). At 24 h post-transfection, the cells were treated with poly (I:C). The cell lysates were prepared at 12 h post-treatment and subjected to luciferase activity assay. **b** Increasing doses of Nsp3-expressing plasmids (0, 0.5, 1.0, and 1.5 μg), pNF-κB-Luc (0.5 μg), and pRL-TK (0.025 μg) were co-transfected into the IPEC-J2 cells and HEK-293 T cells. At 24 h post-transfection, poly(I:C) was added to activate the NF-κB signaling pathway. The cell samples were collected at 36 h post-transfection and subjected to luciferase activity assay. Results are representative of three independent experiments. Data are presented as mean ± SD. *P < 0.05 and **P < 0.01 were considered to be statistically significant and highly significant, respectively

The role of key TGEV proteins involved in the inhibition of NF-κB signaling pathway was evaluated by transfecting the plasmids encoding TGEV proteins into the HEK-293 T and IPEC-J2 cells. The inhibition of NF-κB signaling pathway was assessed using a luciferase reporter assay system. The luciferase reporter analysis indicated that all TGEV proteins, except Nsp2, inhibited the NF-κB signaling pathway to varying extents. Moreover, Nsp1 and Nsp3 were the most potent inhibitors of NF-κB signaling (Fig. 2a). The degree of NF-κB signaling pathway inhibition in the host cell exerted by Nsp3 was evaluated by transfecting the IPEC-J2 and HEK-293 T cells with increasing doses of Nsp3-expressing plasmids. We observed that Nsp3 could dose-dependently suppress the activation of NF-κB signaling pathway (Fig. 2b). These results indicate that Nsp3 plays an important role in the inhibition of NF-κB signaling pathway during TGEV infection.

### Nsp3 inhibits IκBα degradation and restricts p65 nuclear translocation and phosphorylation

**Fig. 3:**
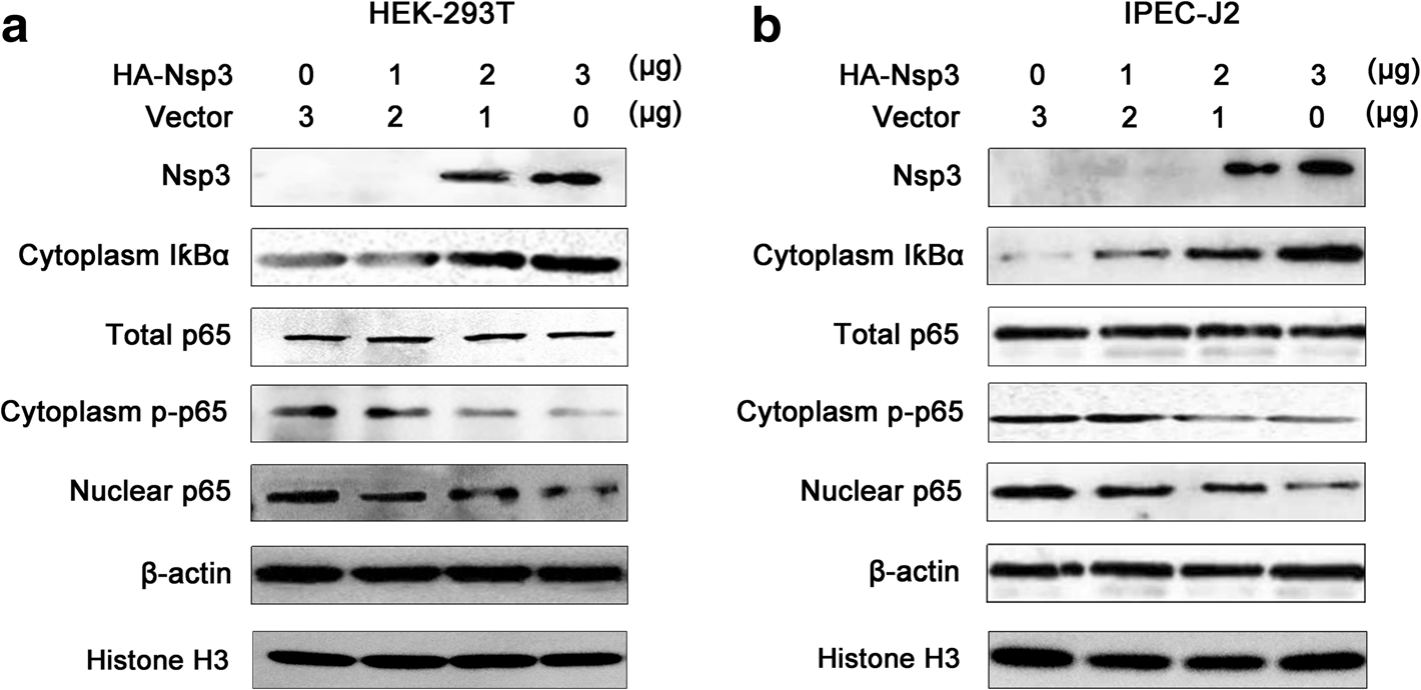
Nsp3 inhibits IκBα degradation, p65 nuclear translocation. **a** HEK-293 T cells or (**b**) IPEC-J2 cells seeded into 24-well plates at a concentration of 0.5–1.0 × 10^5^ cells/mL were co-transfected with different doses of Nsp3-expressing plasmid and different doses of empty vector, which was added to maintain the total transfection amount constant. Poly (I:C) was added at 24 h post-transfection. After 12 h, nuclear proteins and cytoplasmic proteins were extracted to measure the expression of p65, IκBα, and p-p65 by western blotting analysis

NF-κB activation is characterized by the degradation of IκBα as well as the phosphorylation and nuclear translocation of p65 [24]. Therefore, it is important to determine the effect of Nsp3 on IκBα and p65. HEK-293 T (Fig. 3a) and IPEC-J2 (Fig. 3b) cells were transfected with either different titers of Nsp3 or an empty vector. Further, the transfected cells were treated with poly(I:C) for activating NF-κB. The nuclear and cytoplasmic proteins of the cells were extracted and the expression levels of p65, IκBα, and p-p65 were quantified by western blotting. Western blotting analysis revealed that the IκBα expression gradually increased with an increase in the dose of Nsp3-expressing plasmid. Moreover, we observed that Nsp3 had no significant contribution to the total amount of p65. However, the levels of phosphorylated and nuclear p65 decreased with an increase in the Nsp3 levels. These data indicate that Nsp3 inhibits the degradation of IκBα as well as the phosphorylation and nuclear translocation of p65.

### Effect of Nsp3 on NF-κB-regulated cytokine expression

**Fig. 4:**
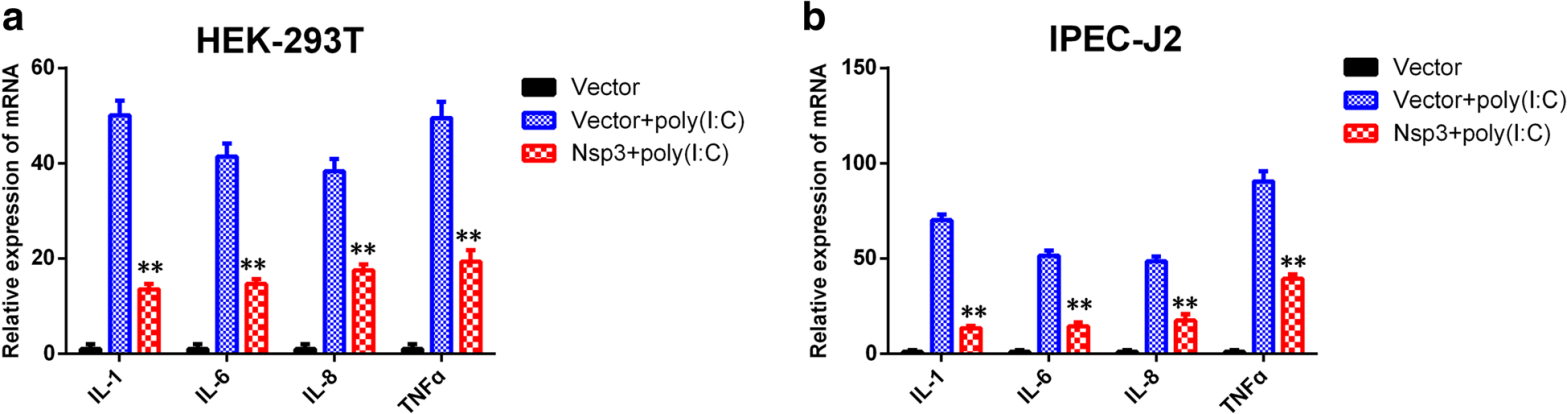
Effect of Nsp3 on NF-κB-regulated cytokine expression. **a** HEK-293 T cells or (**b**) IPEC-J2 cells seeded into 24-well plates at a concentration of 0.5–1.0 × 10^5^ cells/mL were co-transfected with eukaryotic expression plasmid Nsp3 (1 μg) and the empty vector pCMV-HA (1 μg). At 24 h post-transfection, 10 μg/mL poly (I:C) was added to activate the NF-κB signaling pathway, while sterile PBS was used as a negative control. After 12 h, the cell extracts were collected and mRNA levels of IL-1, IL-6, IL-8, TNF-α and β-actin were detected by real-time PCR. β-actin was used as an internal reference gene. Values are the mean ± SD of three independent tests. *P < 0.05 and **P < 0.01 compared with mock infection group

Next, we investigated whether TGEV Nsp3 inhibits NF-κB-mediated cytokine production. HEK-293 T and IPEC-J2 cells were transfected with either Nsp3-expressing plasmid or pCMV-HA vector. The cells were treated with poly(I:C) to induce the activation of NF-κB signaling pathway at 24 h post-transfection. The mRNA levels of IL-1, IL-6, IL-8, TNF-α, and β-actin in cells were quantified using RT-PCR at 12 h post-treatment. The expression levels of IL-1, IL-6, IL-8, and TNF-α in the Nsp3-transfected group were lower when compared to those in the poly(I:C) treatment group (Fig. 3b). These findings demonstrate that Nsp3 inhibits NF-κB-regulated cytokine gene expression by inhibiting the NF-κB signaling pathway in both HEK-293 T (Fig. 4a) and IPEC-J2 (Fig. 4b) cells.

### Amino acid residues at positions 590–1,215 in Nsp3 exert the most potent inhibitory effect on NF-κB signaling

**Fig. 5:**
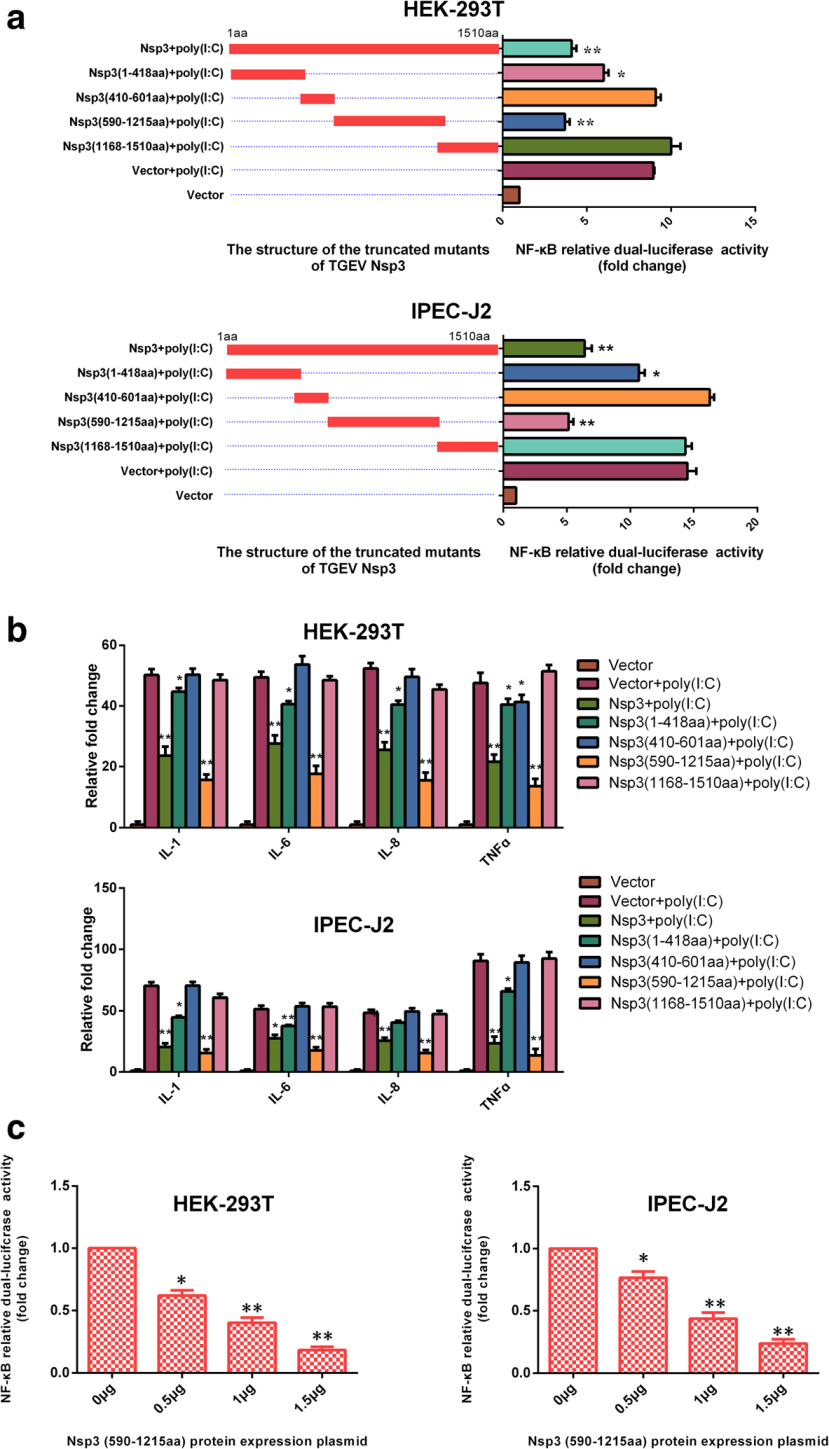
Amino acids at positions 590–1215 in Nsp3 play a vital role in the inhibition of the NF-κB signaling pathway. IPEC-J2 and HEK-293 T cells were co-transfected with pNF-κB-Luc (0.5 μg), pRL-TK (0.025 μg), and one of the following plasmids (0.5 μg): pCMV-HA-Nsp3 (1–418 aa), pCMV-HA-Nsp3 (410–601 aa), pCMV-HA-Nsp3 (590–1215 aa), pCMV-HA-Nsp3 (1168–1510 aa), or pCMV-HA. After 24 h, the cells were treated with poly (I:C), while the cells that were transfected with pCMV-HA were treated with poly (I:C) or PBS as the positive and negative controls, respectively. At 12 h post-treatment, the cell lysates were prepared and subjected to dual luciferase assays (**a**), the RNA was extracted from cells and the mRNA levels of IL-1, IL-6, IL-8, TNF-α and β-actin were real-time PCR (**b**). β-actin was used as an internal reference gene. **c** HEK-293 T and IPEC-J2 cells were co-transfected with pNF-κB-Luc (0.5 μg), pRL-TK (0.025 μg), and different doses of Nsp3 (590–1215 aa) eukaryotic expression plasmids (0, 0.5, 1.0, and 1.5 μg). At 24 h post-transfection, 10 μg/mL poly (I:C) was added to the cells. The cells were harvested and analyzed for luciferase activity at 36 h post-transfection. Data are presented as mean ± SD. *P < 0.05 and **P < 0.01 were considered to be statistically significant and highly significant, respectively

The key functional domains of TGEV Nsp3 involved in the inhibition of NF-κB signaling pathway were examined using expression vectors that encoded truncated Nsp3. The truncated expression vectors were constructed based on the structure of TGEV Nsp3, which was predicted by the online program SMART (http://smart.embl-heidelberg.de/). The IPEC-J2 and HEK-293 T cells were co-transfected with the truncated Nsp3 and the pNF-κB-Luc reporter plasmid. The cells were then treated with poly(I:C) at 24 h post-transcription. Further, luciferase activity and gene expression in the cells were quantified. The luciferase activity analysis revealed that Nsp3 (1–418 aa) and Nsp3 (590–1,215 aa) inhibited the NF-κB signaling pathway activation in both IPEC-J2 and HEK-293 T cells (Fig. 5a). Moreover, RT-PCR analysis indicated that mRNA levels of the NF-κB-related cytokines (IL-1, IL-6, IL-8, and TNF-α) were downregulated upon transfection with Nsp3 (1–418 aa) and Nsp3 (590–1215 aa) plasmids (Fig. 5b). Particularly, Nsp3 (590–1215 aa)-transfected cells exhibited significant attenuation of the NF-κB signaling pathway and expression of NF-κB-regulated cytokines when compared to theother Nsp3 truncated expression vectors(1–418 aa)-transfected cells. The effect of Nsp3 (590–1,215 aa) expression on the inhibition of the NF-κB signaling pathway was examined by co-transfecting pNF-κB-Luc reporter plasmid and different doses of Nsp3 (590–1,215 aa) eukaryotic expression plasmid into the HEK-293 T and IPEC-J2 cells. The Nsp3 (590–1215 aa)-transfected cells exhibited a dose-dependent inhibition of the NF-κB activation (Fig. 5c).

### Nsp3 (590–1,215 aa) inhibits NF-κB signaling by suppressing the IκBα degradation and inhibiting the phosphorylation and nuclear translocation of p65

**Fig. 6:**
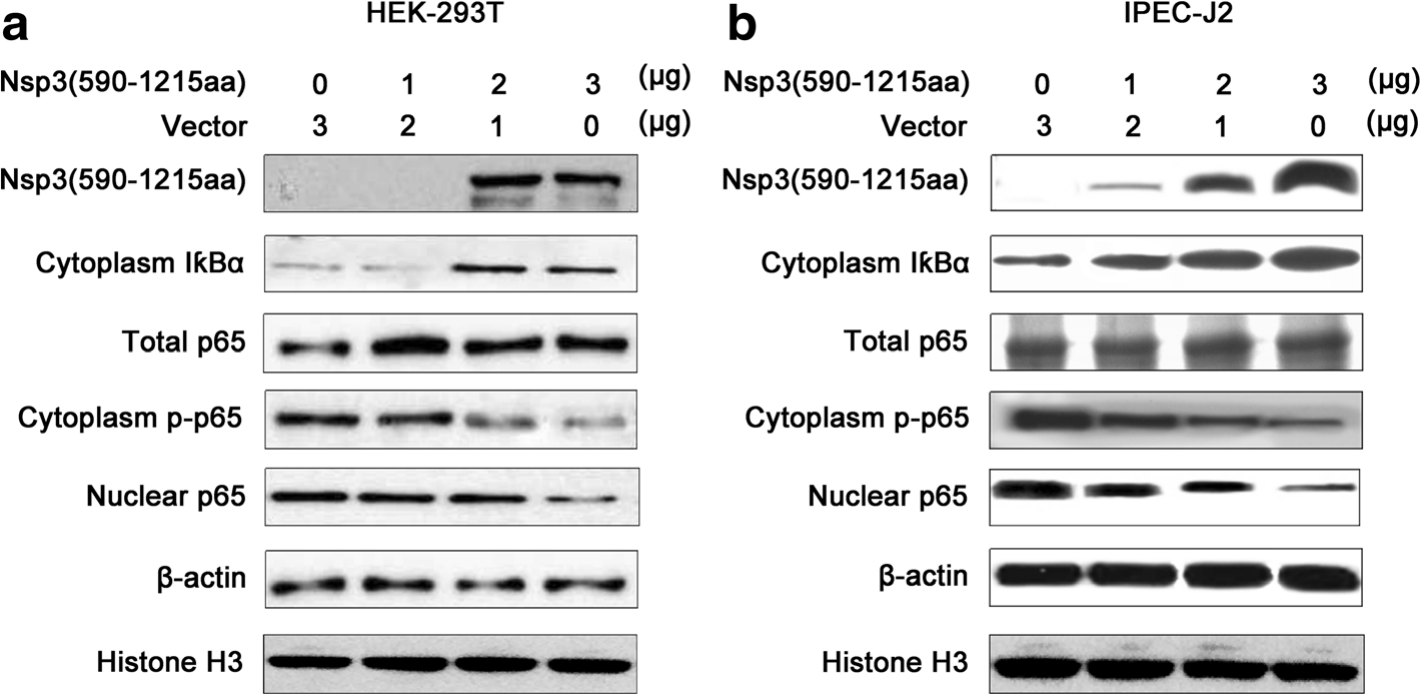
Nsp3 (590–1,215 aa) regulates IκBα degradation and p65 activity. Different doses of eukaryotic expression plasmid Nsp3 (590–1215 aa) were transfected into (**a**) HEK-293 T cells and (**b**) IPEC-J2 cells. The total amount of transfected plasmid of each group was kept consistent by adding different doses of pCMV-HA. After 24 h post-transfection, the cells were treated with poly (I:C). After 12 h, the nuclear and cytoplasmic proteins were extracted, and the expression of p65, IκBα and p-p65 were quantified by western blotting. Results are representative of three independent experiments

The effect of Nsp3 (590–1,215 aa) transfection on the expression of IκBα and p65, which are key proteins in the NF-κB signaling pathway, was investigated using the HEK-293 T and IPEC-J2 cells co-transfected with Nsp3 (590–1,215 aa) eukaryotic expression recombinant plasmid. The co-transfected cells were treated with poly(I:C) to activate the NF-κB pathway. The nuclear and cytoplasmic proteins were extracted at 36 h post-transfection and the expression levels of p65, IκBα, and p-p65 were quantified by western blotting. We observed a gradual increase in the protein expression level of IκBα with an increase in the Nsp3 (590–1,215 aa) protein expression in both HEK-293 T (Fig. 6a) and IPEC-J2 (Fig. 6b) cells, without affecting the total amount of intracellular p65. However, the level of cytoplasmic p-p65 and nuclear p65 decreased with an increase in the Nsp3 (590–1,215 aa) expression level. These results indicate that Nsp3 (590–1,215 aa) dose-dependently inhibits the degradation of IκBα as well as the phosphorylation and nuclear translocation of p65.

### Nsp3 promotes deubiquitination and Nsp3 (590–1,215 aa) inhibits the ubiquitination of IκBα

**Fig. 7:**
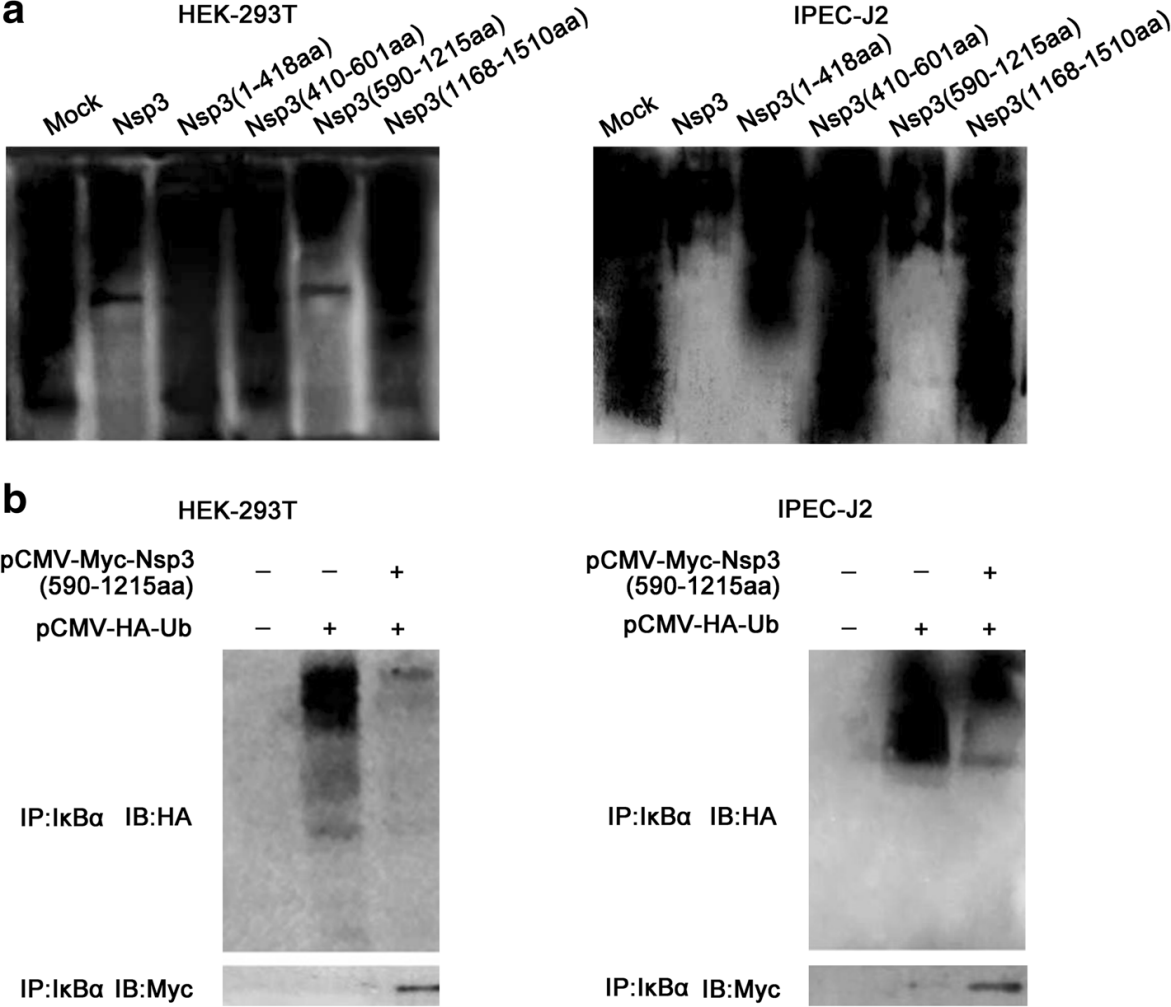
Nsp3 causes deubiquitination and Nsp3 (590–1215 aa) can inhibit the ubiquitination level of IκBα. **a** HA-tagged ubiquitin eukaryotic expression plasmid (pCMV-HA-Ub) and the Myc-tagged Nsp3, or its truncated gene fragments, were co-transfected into the IPEC-J2 cells or HEK-293 T cells. At 24 h post-transfection, the cells were treated with 10 μg/mL poly (I:C). After 12 h, the level of protein ubiquitination in cells was detected by western blotting. **b** IPEC-J2 cells or HEK-293 T cells were co-transfected with pCMV-HA-Ub and Nsp3 (590–1215 aa) eukaryotic expression plasmids. After transfection for 24 h, 10 μg/mL poly (I:C) was added to the cells. After 12 h, the cell lysates were collected for co-immunoprecipitation experiments. Results are representative of three independent experiments

The mechanism underlying the suppressive effects of Nsp3 on the NF-κB signaling pathway was evaluated by co-transfecting the HA-tagged ubiquitin eukaryotic expression plasmid as well as the Myc-tagged Nsp3 and its truncated gene fragments into the IPEC-J2 and HEK-293 T cells. The level of protein ubiquitination levels were quantified by western blotting. The results showed that the high expression of Nsp3, Nsp3 (1–418 aa), and Nsp3 (590–1,215 aa) reduced cellular protein ubiquitination to varying degrees. Moreover, the deubiquitination effect of Nsp3 and Nsp3 (590–1,215 aa) was significantly higher than that of Nsp3 (1–418 aa) in both HEK-293 T and IPEC-J2 cells (Fig. 7a). The effect of Nsp3 (590–1,215 aa) transfection on IκBα ubiquitination was evaluated by transfecting the Nsp3 (590–1,215 aa) and pCMV-HA-Ub eukaryotic expression plasmids into the IPEC-J2 and HEK-293 T cells. The cell extracts were subjected to co-immunoprecipitation. As shown in Fig. 7b, Nsp3 (590–1,215 aa) transfection decreased the IκBα ubiquitination levels. These results indicate that Nsp3 can induce deubiquitination and that the amino acids at positions 590–1,215 in Nsp3 may inhibit the degradation of IκBα by decreasing the IκBα ubiquitination levels, which results in the suppression of NF-κB signaling pathway.

## Discussion

The innate immune response is the first line of host defense against viral infections, which is regulated by diverse signaling pathways. The NF-κB signaling pathway plays a crucial role in the innate immune response regulatory network and is highly active during viral infections. This pathway activates the transcription of genes encoding several cytokines and chemokines, which are involved in the immune response [9]. A variety of viruses evade the host immune response by inhibiting the NF-κB signaling pathway. Earlier studies have reported that SARS-CoV and HCoV-OC43 can impair NF-κB activation [17, 25]. Similarly, the ORF4b-encoded accessory protein (p4b) of MERS-CoV is known to facilitate innate immune evasion by inhibiting the NF-κB signaling pathway [18]. However, knowledge regarding the signal transduction mechanisms of host cells after TGEV infection is incomplete.

Our earlier study revealed that infection with TGEV can activate the NF-κB signaling pathway in both ST and IPEC-J2 cells [21]. In this study, we used poly(I:C), a synthetic analogue of viral double-stranded RNA (dsRNA), to induce an innate immune response via the activation of NF-κB signaling. This strategy enabled the detection of NF-κB signaling pathway inhibition post-TGEV infection. Our results indicated that TGEV infection inhibited NF-κB activity and that this inhibitory effect can be correlated with infection time and inoculum titer in both ST and IPEC-J2 cells.

As proteins are the ultimate performers of biological functions, we examined the TGEV proteins that play an important role in the inhibition of NF-κB signaling. As the transfection efficiency in ST cells was low, we chose HEK-293 T and IPEC-J2 cells for subsequent experiments. The dual luciferase reporter assay analysis revealed that all TGEV proteins, except Nsp2, can inhibit the NF-κB pathway to varying degrees. However, the inhibitory effect of Nsp1 and Nsp3 on NF-κB signaling was higher than that of other TGEV proteins. Further, we explored the underlying mechanisms of Nsp1-mediated NF-κB pathway inhibition. Unfortunately, the expression level of the Nsp1 plasmid was too low in the HEK-293 T and IPEC-J2 cells to complete our analysis. Hence, we only demonstrate the mechanism of action of TGEV Nsp3 in inhibiting the NF-κB pathway.

The multi-domain Nsp3 protein is the largest protein encoded by the coronavirus genome [26]. Several studies have reported that Nsp3 of coronavirus can inhibit multiple signaling pathways. Nsp3 of MHV-A59 can inactivate IRF 3 and consequently inhibit type I IFN response [27]. Similarly, PEDV Nsp3 was reported to be an NF-κB antagonist [20]. SARS Nsp3 is reported to bind to the viral or host cell RNA and regulate the viral replication and evade the immune response of the infected host cell [28]. TGEV is a typical α-type coronavirus. However, the role of TGEV Nsp3 in the regulation of NF-κB signaling is still unclear. In this study, we demonstrated that Nsp3 can inhibit the NF-κB activation and that the inhibitory effect was positively correlated with the expression level of Nsp3.

In most cells, NF-κB complexes are inactive and predominantly reside in the cytoplasm in a complex with inhibitory IκB proteins [9]. Upon activation of the signaling pathways, the IκB protein is degraded and the NF-κB dimers enter the nucleus to modulate the expression of target genes. NF-κB signaling pathway is activated through the canonical or noncanonical pathway and depends on the phosphorylation-induced ubiquitination of IκB proteins [29]. In this study, we evaluated the expression of IκBα and p65 in the HEK-293 T and IPEC-J2 cells transfected with Nsp3. We observed that Nsp3 could dose-dependently inhibit IκBα degradation, as well as the phosphorylation and nuclear translocation of p65. Thus, our data demonstrated that TGEV Nsp3 can suppress the NF-κB signaling through the canonical pathway.

NF-κB signaling is a central pathway that regulates the expression of proinflammatory cytokines [30]. Previous studies have demonstrated that NF-κB is essential in priming inflammasome activation for the production of cytokines, such as TNF-α, IL-1, IL-6, and IL-8 [31, 32]. Several viral proteins play an important role in the regulation of inflammation via NF-κB signaling. The HBeAg protein of HBV suppresses the lipopolysaccharide-induced NLRP3 inflammasome activation and IL-1b production via the inhibition of NF-κB phosphorylation [33]. Similarly, BVDV infection was reported to trigger NF-κB signaling and enhance IL-8 transcription, as the transcription level was observed to be markedly increased after the viral infection, and the immediate early response 3 (IER3) was also reported to inhibit NF-κB activity and downregulate IL-8 expression by approximately 65% [34]. Our experimental results indicated that Nsp3 can downregulate the expression of IL-1, IL-6, IL-8, and TNF-α through the inhibition of NF-κB signaling pathway. These results suggest that Nsp3 plays a vital role in the TGEV-mediated inhibition of the NF-κB signaling pathway.

The domains of Nsp3 involved in the degradation of IκBα and phosphorylation of p65 was examined using the SMART database to predict the possible functional domains of Nsp3. Nsp3 was truncated into four fragments without affecting its original functional domain, and the truncated genes were screened with a dual luciferase assay. The two truncated gene fragments, Nsp3 (1–418 aa) and Nsp3 (590–1,215 aa) strongly inhibited the activation of NF-κB signaling pathway, with the inhibitory effect of Nsp3 (590–1,215 aa) being significantly higher than that of Nsp3 (1–418 aa). The SMART database predicted that both Nsp3 (1–418 aa) and Nsp3 (590–1,215 aa) contain PLPs (154–406 and 606–901 aa). PLPs exhibit deubiquitinating activity, removing the ubiquitin moiety from the signaling molecule in the innate antiviral pathway to inhibit the host innate immunity [26, 35, 36]. Coronavirus PLPs are reported to be the suppressors of innate immune response. SARS-CoV PLP inhibits IFN induction and NF-κB signaling pathways by regulating the activation of important signaling proteins in the IRF3 and NF-κB signaling pathways [37]. Similarly, HCoV-NL63 uses PLPs to evade the innate antiviral response of the host through the inhibition of p53-IRF7-IFNβ signaling [38]. We hypothesized that Nsp3 may utilize the deubiquitination effects of PLP to inhibit the NF-κB signaling pathway by suppressing IκBα ubiquitination. Our results demonstrated that Nsp3 (590–1,215 aa) had markedly deubiquitination activity in the HEK-293 T and IPEC-J2 cells, so Nsp3(590–1,215aa) was used to verify our hypothesis. Consistent with our expected, the amino acid residues at positions 590–1,215 of Nsp3 can inhibit the degradation of IκBα by reducing its ubiquitination level. This phenomenon is likely associated with the deubiquitinating enzyme activity of PLP2, which exists within Nsp3 amino acid residues 606–901.

Previous studies have demonstrated that TGEV infection can activate the NF-κB signaling pathway by inducing the degradation of IκBα, and the degradation of IκBα is mainly caused by ubiquitination [21, 22, 39]. These results indicated that the level of IκBα ubiquitination was not decreased in TGEV-infected cells. Hence, it can be presumed that deubiquitination effects of IκBα that is induced by Nsp3 could not inhibit the activation of NF-κB completely during TGEV infection.

## Conclusions

TGEV infection was demonstrated to inhibit the activation of NF-κB signaling pathway in both ST and IPEC-J2 cells. Moreover, our results indicate that TGEV Nsp3 inhibits NF-κB signaling through the canonical pathway. However, whether Nsp3 simultaneously affects both NF-κB signaling pathway and other pathways warrants further study. The amino acid residues at positions 590–1,215 in Nsp3 have the ability to inhibit the phosphorylation and nuclear translocation of p65 by inhibiting the ubiquitination of IκBα. We speculate that this may be due to the presence of a PLP2 domain with deubiquitinating enzyme activity at amino acid residue positions 590–1,215 in Nsp3. Our study provides a better understanding of the TGEV-mediated innate immune modulation and lays the basis for future studies on the pathogenesis of coronavirus.

## Data Availability

All data generated or analyzed during this study are included in this published article.

## Abbreviations

3CLpro: 3C-like protease
DMEM: Dulbecco’s modified Eagle’s medium
dsRNA: Double-stranded RNA
FBS: Fetal bovine serum
IFN: Interferon
IKK: IκB kinase
IL: Interleukin
IPEC-J2: Intestinal epithelial cell lines J2
IRF3: Interferon regulatory factor 3
IκB: Inhibitor of nuclear factor kappa-B
Luc: Luciferase reporter gene
mAbs: Monoclonal antibodies
MOI: Multiple of infection
NF-κB: Nuclear factor-kappa B
Nsp: Non-structural protein
ORFs: Open reading frames
PBS: Phosphate-buffered saline
PLP: Papain-like protease
p-p65: Phosphorylated p65
RT-PCR: Quantitative real-time PCR
ST: Swine testicular
TGEV: Transmissible gastroenteritis virus
TNF-α: Tumor necrosis factor α
UTR: Untranslated region
